# Utility of under-sampled scans with iterative reconstruction and high-frequency preserving transform for high spatial resolution magnetic resonance cholangiopancreatography

**DOI:** 10.1007/s11604-024-01688-z

**Published:** 2024-11-05

**Authors:** Shota Kondo, Yuko Nakamura, Toru Higaki, Takashi Nishihara, Masahiro Takizawa, Toru Shirai, Motoshi Fujimori, Yoshitaka Bito, Keigo Narita, Dara Fonseca, Shogo Maeda, Ikuo Kawashita, Yukiko Honda, Kazuo Awai

**Affiliations:** 1 Diagnostic Radiology, Hiroshima University, 1-2-3 Kasumi, Minami-Ku, Hiroshima City, Hiroshima 734-8551 Japan; 2 Graduate School of Advanced Science and Engineering, Hiroshima University, 1-4-1 Kagamiyama, Higashi-Hiroshima City, Hiroshima 739-8527 Japan; 3 FUJIFILM Corporation, 2-1, Shintoyofuta, Kashiwa City, Chiba 277-0804 Japan; 4 Present Address: Department of Diagnostic Imaging, Hokkaido University Graduate School of Medicine, Kita 15 jo, Nishi 7 chome, Kita ku, Sapporo City 060-8638, Japan

**Keywords:** Under-sampled scans, Iterative reconstruction, High-frequency preserving transform, Spatial resolution, Magnetic resonance cholangiopancreatography

## Abstract

**Purpose:**

Under-sampled scans with iterative reconstruction and high-frequency preserving transform (Us-IRHF) can increase the acquisition speed without degrading the image quality by recovering image information from under-sampled data. We investigate the clinical applicability of high spatial resolution magnetic resonance cholangiopancreatography (MRCP) images without extending the scanning time using Us-IRHF.

**Methods:**

A slit phantom was scanned with conventional- (without Us-IRHF), Us-IR- (without HF), and Us-IRHF scanning. The matrix size was 320 × 320 for Us-IR- and Us-IRHF- and 288 × 208 for conventional scanning. Modulation transfer function (MTF) focused on the 1.0 lp/cm gauge for each scanning was calculated. For clinical study we acquired respiratory-triggered 3D MRCP scans with and without Us-IRHF (U^+^-, U^−^MRCP) in 41 patients. The matrix size was 320 × 320 for U^+^- and 288 × 208 for U^−^MRCP. The acquisition time and the relative duct-to-periductal contrast ratios (RCs) for the right- and left intrahepatic bile-, the common bile-, and the main pancreatic duct were recorded. Visualization of each duct and overall image quality was scored on 5-point confidence scales. For visualization of each duct the score ranged from 1 (not visible) to 5 (visible with excellent details), for the image quality, it ranged from 1 (undiagnostic) to 5 (excellent). Superiority for the qualitative visualization score and non-inferiority for the RC values with prespecified margins were assessed.

**Results:**

Phantom study showed that compared to the conventional- and Us-IR (without HF) images, the MTF for the Us-IRHF image revealed the highest response. For clinical study, the mean acquisition time was 161 s for U^+^- and 165 s for U^−^MRCP. For all ducts, the RC value of U^+^MRCP was non-inferior to U^−^MRCP and the qualitative visualization score assigned to U^+^MRCP was superior to U^−^MRCP.

**Conclusion:**

Us-IRHF improved the image quality of high spatial resolution MRCP without extending the scanning time.

**Supplementary Information:**

The online version contains supplementary material available at 10.1007/s11604-024-01688-z.

## Introduction

Endoscopic retrograde cholangiopancreatography (ERCP) has been considered the diagnostic reference standard for the evaluation of a wide range of abnormalities involving the pancreatobiliary tree, including choledocholithiasis, structuring diseases and duct leaks/disruptions [1, 2]. ERCP is invasive and requires sedation, analgesia, and ionizing radiation; it may elicit post-procedural problems such as pancreatitis, bleeding, and pain [3, 4]. Due to the availability of noninvasive cross-section imaging techniques such as abdominal ultrasound (US), computed tomography (CT), endoscopic ultrasound (EUS), and magnetic resonance cholangiopancreatography (MRCP), it is now an almost exclusively therapeutic—rather than diagnostic procedure [5, 6].

MRCP can demonstrate the pancreatobiliary duct system noninvasively without sedation, analgesia, and ionizing radiation and it poses no risk of post-procedural problems [7, 8]. Unlike ERCP, MRCP also allows direct visualization of the hepatic and pancreatic parenchyma and surrounding structures. However, as the sensitivity for anatomic details was lower on MRCP- than ERCP images, it was not useful for a management algorithm addressing stone- or non-stone diseases of the biliary tree or pancreas [4, 9]. Consequently, for the replacement of ERCP with MRCP as a diagnostic method, the image quality of MRCP must be improved.

MRCP images with high spatial resolution can be obtained with the respiratory-triggered technique. However, as it involves a long scanning time, artifacts due to physiologic motion may increase [10, 11]. The compressed sensing (CS) technique attempts to increase the acquisition speed by recovering image information from highly under-sampled sparse k-space data [12]. Others [13] focused on the utility of CS for reducing the acquisition time without degrading the image quality. However, anatomic details may be degraded with CS due to slight blurring, a specific imaging artifact elicited by the denoising effect [13–15]. Under-sampled scans with iterative reconstruction and high-frequency preserving transform (Us-IRHF) apply an under-sampled algorithm using Golden Angle in the ky–kz plane and image-quality degradation due to under-sampling can be improved with an iterative reconstruction employing regularization with the wavelet transform and a high-frequency preserving transform. Unlike CS, the combination of iterative reconstruction and high-frequency preserving transform can improve the image quality and avoid the degradation of anatomic details because high frequency components are maintained with a high-frequency preserving transform. We investigated the clinical applicability of Us-IRHF for high spatial resolution MRCP images without extending the scanning time.

## Materials and methods

The quantitative evaluation of the spatial resolution is difficult in clinical studies. Thus, our investigation included a phantom study where the spatial resolution, such as modulation transfer function (MTF), can be evaluated quantitatively and a clinical study for the investigation of the clinical utility of Us-IRHF.

### Under-sampled scans with iterative reconstruction and high-frequency preserving transform

Us-IRHF represents an under-sampled algorithm that applies the Golden Angle in the ky-kz plane. The radial lines are very evenly spaced with time in the ky-kz plane and low frequencies are high densely sampled. The combination of an iterative reconstruction using regularization with the wavelet transform and the high-frequency preserving transform can decrease the degradation of image quality due to under-sampling. Anatomic details can be maintained especially with the high-frequency preserving transform.

#### Principle of Us-IRHF

**Fig. 1:**
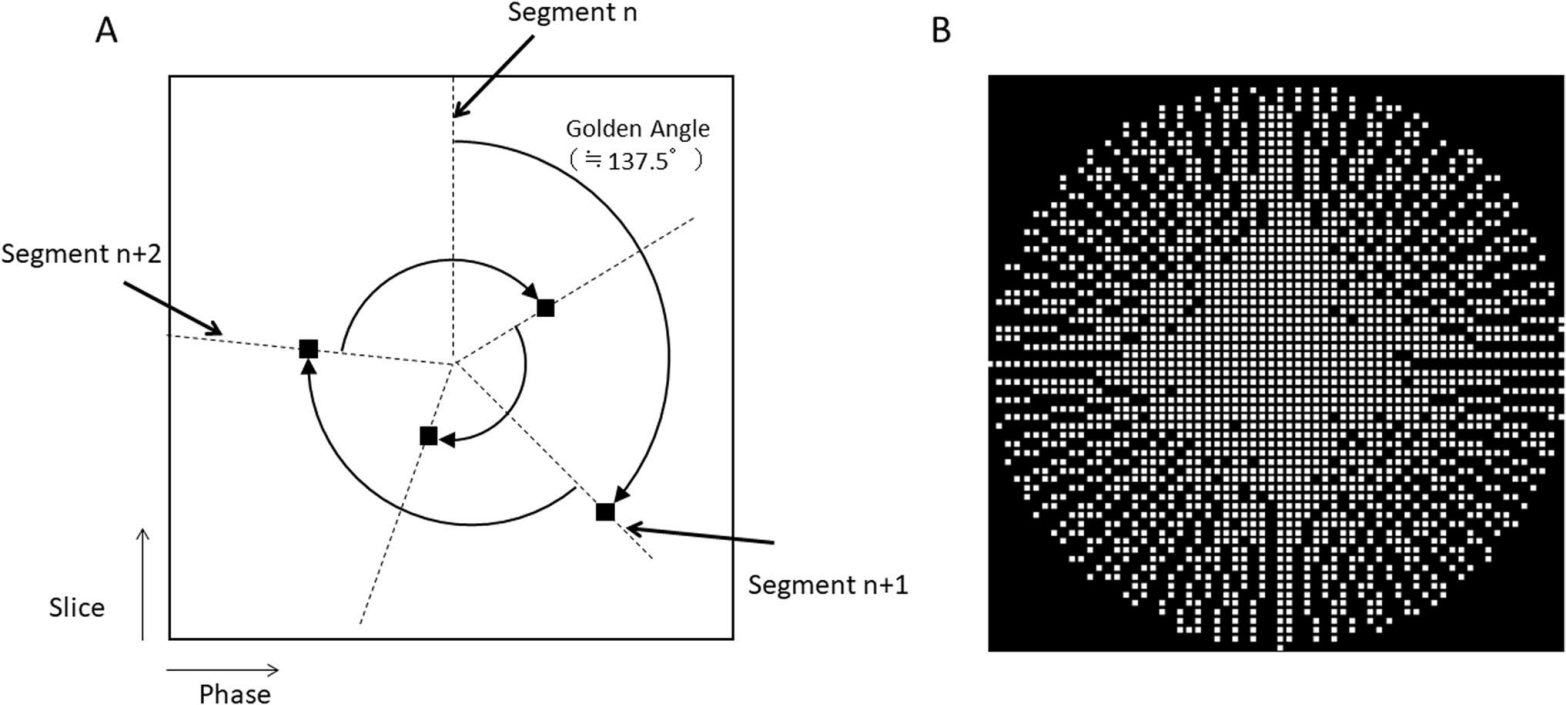
**A** Schematic diagram of Golden Angle radial sampling. **B** Golden Angle radial sampling pattern

Random sampling that samples k-space data randomly in the slice-phase-encoding direction is performed in CS [12]; it includes variable density random sampling [16] and Poisson disk sampling [17, 18]. We used hybrid under-sampling, an equally spaced under-sampling pattern in the phase-encoding direction as is applied in parallel imaging and Golden Angle radial sampling from the outside to the inside of the k-space in the slice phase-encoding direction. Low frequencies are high densely sampled in the slice-phase-encoding direction (Fig. 1).

Under-sampled data using the Golden Angle radial sampling pattern was reconstructed based on the split-Bregman method [19]. The minimized equation where y is the computed data, x the data obtained from imaging, Ψ_w_ the wavelet transform, Ψ_h_ the high-frequency preserving transform that consists of a low-frequency pass filter, λ_1_ and λ_2_ are the coefficients of normalization, and i represents a coil element.

1 $${{\varvec{E}}_{{\varvec{i}}} \left( {\mathbf{x}} \right) = \parallel{\varvec{y}}_{{\varvec{i}}} - {\varvec{x}}_{{\varvec{i}}}\parallel_2^2 + {\varvec{\lambda}}_{1} \,\parallel{{\varvec{\Psi}}}_{{\mathbf{w}}} {\varvec{x}}_{{{\varvec{i}}}}\parallel_1 + {\varvec{\lambda}}_{2} \parallel{{\varvec{\Psi}}}_{{\mathbf{h}}} {\varvec{x}}_{{{\varvec{i}}}}}\parallel_1$$

**Fig. 2:**
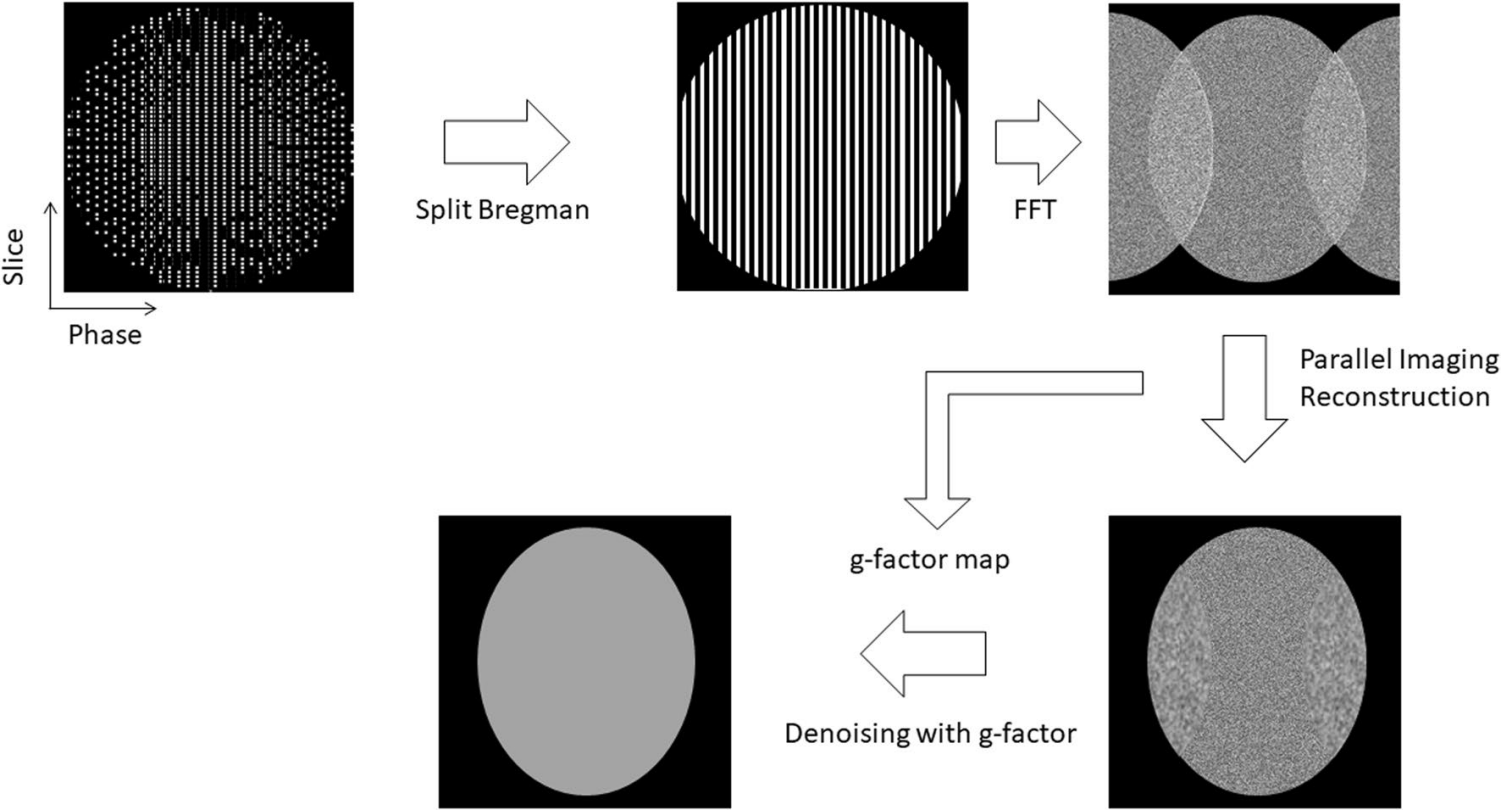
Schematic diagram of the proposed reconstruction method

The high-frequency preserving transform consists of low-pass filtering in the k-space; it is ‘1’ in a tile whose length is half of the k-space domain and it is ‘0’ outside the tile. Its regularization term has less effect on high-frequency than low-frequency components. Therefore, it preserves fine structures such as line-like shapes during minimization and avoids the degradation of anatomic details [16]. Both parameters λ_1_ and λ_2_ were set as 0.5. After the reconstruction of each coil element, parallel imaging reconstruction and wavelet denoising with geometry factor-weighting were applied [20] (Fig. 2).

### Phantom study

Unlike CS, Us-IRHF avoids degrading anatomic details using the high-frequency preserving transform. To examine the effect of Us-IRHF, we performed phantom experiments and compared the results obtained with conventional scanning (without Us-IRHF), scanning with Us-IR (without high-frequency preserving transform [HF]), and with Us-IRHF.

We created a slit phantom featuring a 1.0-, 1.7-, 2.5-, 3.3-, 5.0-, 6.7-, and 10.0 line-pairs per centimeter (lp/cm) gauge that corresponded to a gap size of 5.0-, 3.0-, 2.0-, 1.5-, 1.0-, 0.75-, and 0.5 mm, respectively (Supplementary Fig. 1). This phantom was scanned with a 3 T MRI scanner (TRILLIUM OVAL; FUJIFILM Corporation, Tokyo, Japan) using a 28-channel coil. The gauges were filled with saline and the area around the gauges was filled with an NiCl_2_ solution.

The imaging parameters for conventional scanning (without Us-IRHF) were TR/TE 3333.3/428.4 ms, flip angle 90°, echo train length 115, number of excitations 1, field-of-view (FOV) 280 mm, slice thickness 1.8 mm, number of slices 75, parallel imaging factor 3.0 (phase direction 3.0, slice direction 1.0), matrix size 288 × 208, and Cartesian sampling. Except for the matrix size (320 × 320) and applying Us-IRHF (Golden Angle radial sampling), the imaging parameters for the scanning with Us-IRHF were as for conventional scanning (without Us-IRHF). Undersampling factor used in this study was 1.7, indicating that the percentage reduction in the number of samplings was about 60% (1/1.7). Too high undersampling factor may degrade the image quality. Thus, we determined the highest undersampling factor with maintaining the image quality based on the preliminary data using images of 10 patients not included in this study. Scanning with Us-IR (without HF), i.e. λ_1_ = 1, λ_2_ = 0, was with the imaging parameters applied on the scanning with Us-IRHF.

#### Image analysis

Images were analyzed by a board-certified radiologist (SK with 7 years of experience in radiology) using ImageJ software (http://rsb.info.nih.gov/ij/). The reader evaluated the delineation of each gauge by recording whether or not it was observed separately.

The spatial resolution was evaluated quantitatively using a profile curve and the MTF focused on the 1.0 lp/cm gauge. We calculated the relative signal intensity (SI) using the equation:

$${\text{relative}}\,{\text{SI}}\, = \,{{{\text{SI}}_{{\text{T}}} } \mathord{\left/ {\vphantom {{{\text{SI}}_{{\text{T}}} } {{\text{SI}}_{{\text{B}}} }}} \right. \kern-0pt} {{\text{SI}}_{{\text{B}}} }},$$

where SI_T_ is the SI of the target and SI_B_ is the SI of the background (the area around the gauges filled with NiCl_2_ solution). A profile curve of the relative SI of the 1.0 lp/cm gauge was generated along a horizontal line crossing through the center of the gauge. The MTF, determined with the edge method [21], was calculated as the index for spatial resolution. The edge of the gauge on the image was used to generate the edge-spread function which was then differentiated into a line-spread function and transformed into an MTF using standard Fourier methods.

### Clinical study

This retrospective and observational study was approved by our institutional review board; prior informed consent from participants was waived. Patient records and information were anonymized and de-identified prior to analysis.

#### Study population

The study included 41 patients who had undergone MRCP between January and March 2021. They were 20 men and 21 women (age range 48–89 years; median age 72 years). Of these, 23 underwent MRCP for a follow-up or work-up for pancreatic cystic neoplasms, 5 for chronic pancreatitis, 3 for biliary stones, 3 for autoimmune pancreatitis, 5 for biliary or pancreatic dilation, and 2 for a biliary duct tumor.

#### Image acquisition

All imaging studies were performed on a 3 T MRI scanner (TRILLIUM OVAL; Tokyo, Japan); MRCP scanning was with the respiratory-triggered technique. The parameters for conventional MRCP imaging were TR/TE variable depending on the respiratory rate (range: 2400–5000 ms) /428.4 ms, flip angle 90°, echo train length 115, number of excitations 1, FOV 280 mm, slice thickness 1.8 mm, number of slices 75, parallel imaging factor 3.0 (phase direction 3.0, slice direction 1.0), and Cartesian sampling. Except for the matrix size and applying Us-IRHF (Golden Angle radial sampling and undersampling factor 1.7), the parameters for MRCP scanning with Us-IRHF (U^+^MRCP) were as for scanning without Us-IRHF (U^−^MRCP). The matrix size for U^−^MRCP and U^+^MRCP was 288 × 208 and 320 × 320, respectively. We defined the matrix size for U^+^MRCP as 320 × 320 because our pilot study showed that a matrix size larger than 320 × 320 for U^+^MRCP increased the scanning time. We did not acquire scans with Us-IR (without HF) because it was not possible on our clinical scanner.

#### Image analysis

Quantitative analysis was by one radiologist (SK with 7 years of experience in radiology). For SI measurements, regions of interest (ROIs) were placed on the main pancreatic duct of the pancreatic body (MPD), the common bile duct (CBD) at the level just below the confluence of the cystic and common hepatic duct, the left hepatic duct (LHD), the right hepatic duct (RHD), and on peribiliary- and peripancreatic ductal tissue. The SI was recorded as the mean measurement value of the ROIs. The same radiologist calculated the relative duct-to-periductal contrast ratios (RC) using the equation RC = (SI_duct_-SI_periduct_)/(SI_duct_ + SI_periduct_) [22].

The image quality was independently evaluated by three board-certified radiologists (KN, YN and KA with 8, 21 and 38 years, respectively, of experience in radiology) using the Likert scale [23, 24]. They received standardized instructions and were trained on image sets from five patients not included in this study. They graded the visualization of the pancreaticobiliary duct and the overall image quality. Visualization of the pancreaticobiliary ducts (MPD, CBD, LHD and RHD) was graded using the 5-point Likert scale where 1 = ductal structure not visible, 2 = ductal structure barely identified, 3 = moderate visualization-, 4 = good visualization-, and 5 = excellent visualization of the ductal structure. Due to the higher spatial resolution and application of high frequency preserving transform, we expected the ductal margins to be clearly visualized and without blurring on U^+^MRCP images. The overall image quality was graded using the 5-point Likert scale where 1 = undiagnostic (severe blurring, diagnostic information impaired), 2 = poor (moderate blurring, limited diagnostic information), 3 = fair (some blurring, acceptable diagnostic information), 4 = good (mild blurring, sufficient diagnostic information), and 5 = excellent (minimal or no blurring, excellent diagnostic information) [13, 15, 25]. In the absence of interobserver agreement, final decisions were reached by consensus.

### Statistical analysis

We based our study on the hypothesis that the quality of U^+^MRCP images would be superior to the quality of U^−^MRCP image. Thus, superiority of U^+^MRCP- compared to U^−^MRCP images was assessed for qualitative evaluation. We considered U^+^MRCP images to be superior to U^−^MRCP images when the entire two-sided 95% confidence interval (CI) for the difference between U^+^MRCP- and U^−^MRCP images was larger than 0. Slight blurring, a specific imaging artifact caused by the denoising effect of CS, reduced the RC on MRCP scans [13–15]. Thus, based on the hypothesis that the RC on U^+^MRCP images would be non-inferior to that on U^−^MRCP image non-inferiority of U^+^MRCP- vis-à-vis U^−^MRCP images was assessed for evaluation of the RC. Non-inferiority was recorded when the entire two-sided 95% CI for the difference between U^+^MRCP- and U^−^MRCP images was above the pre-specified non-inferiority margin. The margin was selected as the standard deviation on U^−^MRCP images because we considered a difference smaller than the standard deviation to be clinically negligible. Thus, the prespecified non-inferiority margin (the standard deviation on U^−^MRCP images) was set at 0.07, 0.06, 0.07, and 0.11 for the RC values of the MPD, CBD, LHD, and RHD, respectively.

For qualitative analysis we calculated the interobserver agreement of three readers (KN, YN and KA) using the weighted kappa statistic. A kappa statistic from 0.81 to 1.00 was interpreted as excellent-, from 0.61 to 0.80 as substantial-, from 0.41 to 0.60 as moderate-, from 0.21 to 0.40 as fair-, and from 0.00 to 0.20 as poor agreement [26]. Differences in the acquisition time between U^+^MRCP and U^−^MRCP were determined using the two-sided Wilcoxon signed-rank test.

## Results

### Phantom study

The phantom images are shown in Supplementary Figs. 2 and 3. The 1.0-, 1.7-, 2.5-, and 3.3 lp/cm gauges were observed separately while the 6.7, and 10.0 lp/cm gauges were not on any of the images. The 5.0 lp/cm gauge was visually separated on Us-IR- and Us-IRHF- images but not on conventional images without Us-IRHF. Among the three images, the 5.0 lp/cm gauge margin was separated most clearly on the Us-IRHF image. The profile curve for the 1.0 lp/cm gauge is shown in Supplementary Fig. 4A. The slope of the curve was sharper on the image with Us-IR (without HF) than on the conventional image. The slope of the Us-IRHF image was the sharpest. Compared to the conventional- and Us-IR (without HF) images, the MTF for the Us-IRHF image revealed the highest response (Supplementary Fig. 4B). The acquisition time (161 s) was the same for all images.

### Clinical study

The median acquisition time for U^−^MRCP- and U^+^MRCP scans was 161 s (range 115–297 s) and 158 s (range, 122–211 s), respectively. There was no significant difference between the acquisition times (*p* = 0.28).

**Fig. 3:**
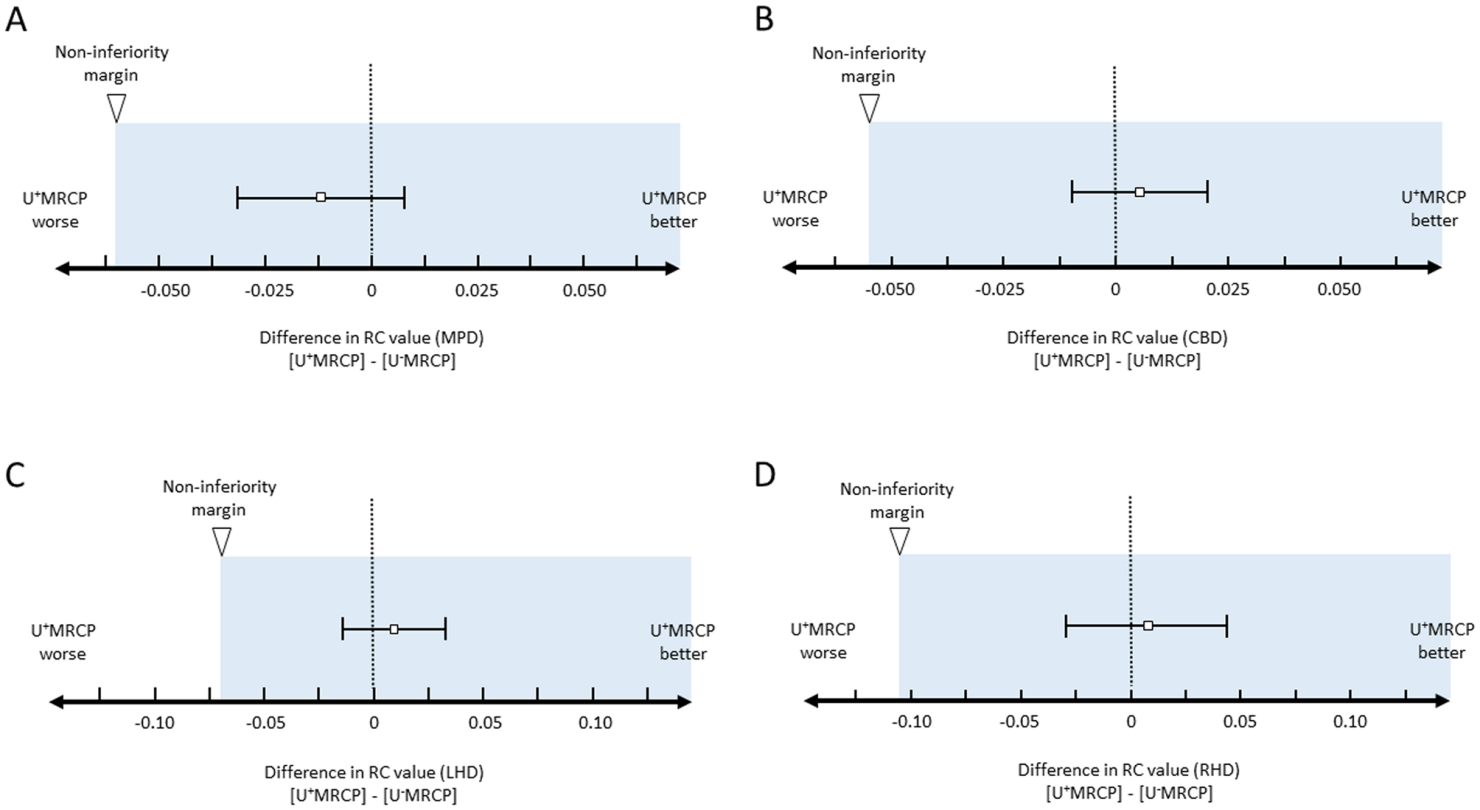
Non-inferiority of RC values on U^+^MRCP- vis-à-vis U^−^MRCP images (**A** MPD, **B** CBD, **C** LHD, and **D** RHD). The RC values for all ducts on U^+^MRCP images were non-inferior to U^−^MRCP images

The RC value of each pancreaticobiliary duct on U^+^MRCP images was comparable to U^−^MRCP images (Table 1). The 95% CI for the difference was −0.032 to 0.010 (MPD), −0.012 to 0.023 (CBD), −0.014 to 0.032 (LHD), and −0.028 to 0.042 (RHD) (Fig. 3A–D).

**Table 1:** RC values and qualitative scores on U^+^MRCP and U^−^MRCP images

	U^+^MRCP	U^−^MRCP
RC values		
MPD	0.80 (0.34–0.93)	0.80 (0.23–0.93)
CBD	0.89 (0.60–0.95)	0.89 (0.56–0.95)
LHD	0.88 (0.63–0.96)	0.88 (0.53–0.96)
RHD	0.86 (0.43–0.94)	0.86 (0.34–0.95)
Image quality scores^*^		
MPD	3.4 (1.1)	3.1 (0.9)
CBD	3.9 (0.9)	3.6 (0.8)
LHD	3.6 (0.9)	3.3 (0.9)
RHD	3.6 (1.0)	3.2 (1.0)
Overall image quality	3.8 (0.9)	3.4 (0.8)

Unless otherwise indicated, data are the median with ranges in parenthesis

*RC* relative duct-to-periductal contrast ratios, *MPD* main pancreatic duct, *CBD* common bile duct, *LHD* left hepatic duct, *RHD* right hepatic duct

^*^Data are the mean (standard deviation)

**Fig. 4:**
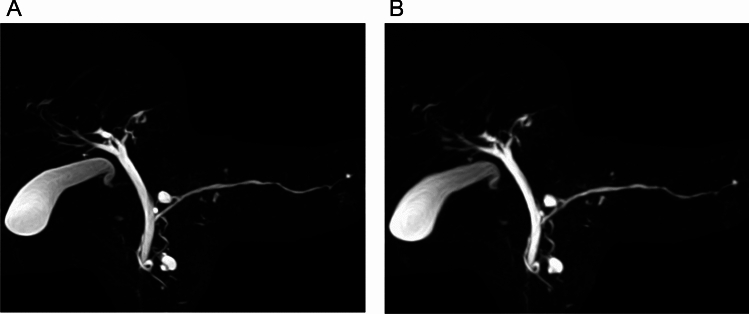
A 73-year-old man with branch-duct intraductal papillary mucinous neoplasms of pancreas. Coronal maximum intensity projection (MIP) images of U^+^MRCP- (**A**) and U^−^MRCP scans (**B**) are shown. The visibility of the pancreaticobiliary ducts and pancreatic cysts are better and the edge of the lesions were sharper on (**A**) than (**B**)

**Fig. 5:**
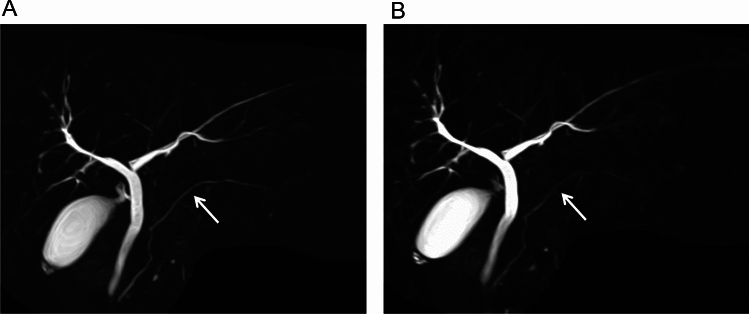
A 56-year-old woman with chronic pancreatitis. Coronal maximum intensity projection (MIP) images of U^+^MRCP- (**A**) and U^−^MRCP scans (**B**) are shown. The main pancreatic duct is more clearly visualized on (**A**) than (**B**)

**Fig. 6:**
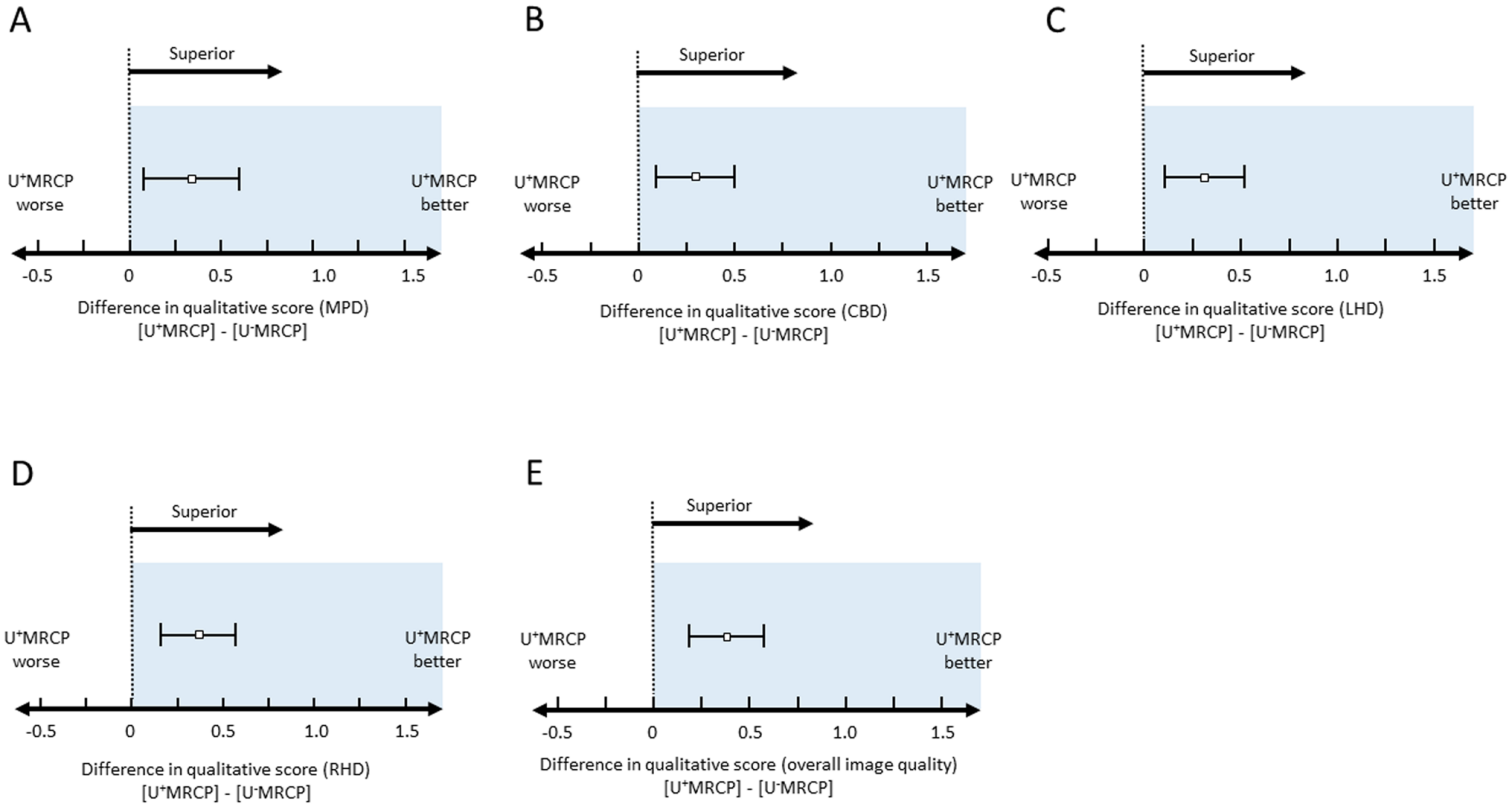
Superiority of the qualitative scores of U^+^MRCP- vis-à-vis U^−^MRCP images (**A** MPD, **B** CBD, **C** LHD, **D** RHD, **E** overall image quality). Qualitative scores for all ducts and the overall image quality of the U^+^MRCP- were superior to the U^−^MRCP images

The qualitative visualization scores for MPD, CBD, LHD, RHD, and the overall image quality for U^+^MRCP- were superior to that of U^−^MRCP images (Figs. 4, 5, 6, and Table 1). The 95% CI for the difference was 0.04 to 0.59 (MPD), 0.08 to 0.50 (CBD), 0.10 to 0.53 (LHD), 0.18 to 0.64 (RHD), and 0.20 to 0.67 (overall image quality).

Interobserver agreement among the three radiologists was substantial (kappa value range 0.65–0.70).

## Discussion

Our clinical study showed that the qualitative visualization score of U^+^MRCP images (high spatial resolution MRCP with Us-IRHF) was superior to U^−^MRCP images (conventional MRCP without Us-IRHF) for all ducts; the overall image quality score for U^+^MRCP- was also superior to U^−^MRCP images. There was no significant difference in the acquisition times. Our phantom study showed that the 5.0 lp/cm gauge was visually separate on the image with Us-IR (without HF)- and with Us-IRHF but not on the conventional image. The margin of the 5.0 lp/cm gauge was separated most clearly on the Us-IRHF image. Based on the result of the profile curve and the MTF, the highest spatial resolution was obtained with the Us-IRHF rather than with Us-IR- and conventional imaging. This indicates that not only increasing the matrix size but also applying HF increased the spatial resolution. Taken together, our Us-IRHF method improved the quality of high spatial resolution MRCP images without extending the scanning time.

Slight blurring, a specific imaging artifact due to the denoising effect of CS, reduced the RC value on MRCP images [13–15]. Our technique may also blur the structure because of the insufficient restoration of sparsely sampled high-frequency regions and the noise reduction technique. Our clinical study revealed that with respect to all ducts, the RC value on U^+^MRCP- was non-inferior to the RC on U^−^MRCP images and our phantom study showed that adding HF to Us-IR scans improved the spatial resolution. We suspect that blurring could be avoided by increasing the matrix size and applying HF to Us-IR scans.

The k-space filling method is Cartesian- for U^−^MRCP and radial sampling for U^+^MRCP scanning. Radial sampling is motion-insensitive because of high densely sampling in low frequencies [27], indicating that the difference in sampling may also improve the quality on U^+^MRCP- compared with U^−^MRCP images. On the other hand, radial sampling may degrade in the spatial resolution because of low densely sampling in high frequencies [28, 29]. Thus, we supposed that Us-IRHF, combination of radial sampling and HF is useful especially when high spatial resolution images are required.

The application of Us-IRHF to MRCP scans can be used to reduce the acquisition time. Although there is a trade-off with respect to the image quality and the acquisition time even when techniques like CS are applied [13, 15], it may be advisable to shorten the acquisition time when scanning patients with unstable breathing because longer scanning times are often associated with a poorer image quality due to irregular breathing [30]. Whether Us-IRHF method can be used to improve the image quality or to reduce the acquisition time must be decided on a case-by-case basis. This topic requires further investigation.

Recently, deep learning (DL)-based approaches which aims at improving the image quality after the rapid reconstruction of under-sampled MRI k-space data has been developed [31, 32]. The combination of Us-IRHF and DL may improve the image quality of high spatial resolution MRCP images with reduced scanning time. However, DL technology is not currently available at our institute.

Our study has some limitations. The study population was relatively small and our investigation was retrospective and conducted at a single institution. Therefore, our findings are preliminary. We did not fully evaluate the diagnostic performance of high spatial resolution MRCP scanning with Us-IRHF because our study subjects were heterogenic of different clinical backgrounds. A more detailed clinical study, investigating the accuracy and reliability of the proposed method in exploring lesion detection and characterization in a detailed disease patient population, would be interesting to pursue in the future. As ERCP has been the diagnostic reference standard for the evaluation of a wide range of abnormalities involving the pancreatobiliary tree [1, 2], studies that compare ERCP with our scanning methods are needed. However, ERCP has not been performed for patients in our study because ERCP should be performed for the purposes of treatment (drainage), but are not the first choice for diagnostic purposes [33].

In conclusion, Us-IRHF can improve the image quality of high spatial-resolution MRCP images without extending the scanning time.

## Supplementary Information

Below is the link to the electronic supplementary material.

Supplementary file 1—Supplementary Fig. 1 Design (A) and actual photo (B) of our slit phantom. The gauges were filled with saline and the area around the gauges was filled with an NiCl2 solution

Supplementary file 2—Supplementary Fig. 2 Phantom images obtained with conventional- (A), Us-IR- (without high-frequency preserving transform) (B), and Us-IRHF scanning (with high-frequency preserving transform) (C). The margin of the 5.0 lp/cm gauge (arrows) was separated visually on (B) and (C), but not on (A)

Supplementary file 3—Supplementary Fig. 3 Magnification (A–C) of images shown in Supplementary Fig. 2. The margin of the 5.0 lp/cm gauge (arrows) was separated most clearly on (C)

Supplementary file 4—Supplementary Fig. 4 Profile curve (A) and MTF (B) for the 1.0 lp/cm gauge obtained with conventional-, Us-IR- and Us-IRHF image

Supplementary file 5—Supplementary Table 1 Qualitative scores on U^+^MRCP and U^-^MRCP images of each of three readers

## References

[1] McCune WS, Shorb PE, Moscovitz H. Endoscopic cannulation of the ampulla of vater: a preliminary report. Ann Surg. 1968;167(5):752–6. 5646296 10.1097/00000658-196805000-00013 PMC1387128

[2] Adler DG, Baron TH, Davila RE, Egan J, Hirota WK, Leighton JA, et al. ASGE guideline: the role of ERCP in diseases of the biliary tract and the pancreas. Gastrointest Endosc. 2005;62(1):1–8. 15990812 10.1016/j.gie.2005.04.015

[3] Freeman ML. Complications of endoscopic retrograde cholangiopancreatography: avoidance and management. Gastrointest Endosc Clin N Am. 2012;22(3):567–86. 22748249 10.1016/j.giec.2012.05.001

[4] Dillman JR, Patel RM, Lin TK, Towbin AJ, Trout AT. Diagnostic performance of magnetic resonance cholangiopancreatography (MRCP) versus endoscopic retrograde cholangiopancreatography (ERCP) in the pediatric population: a clinical effectiveness study. Abdom Radiol (NY). 2019;44(7):2377–83. 30874847 10.1007/s00261-019-01975-8

[5] Bor R, Madácsy L, Fábián A, Szepes A, Szepes Z. Endoscopic retrograde pancreatography: when should we do it? World J Gastrointest Endosc. 2015;7(11):1023. 26322155 10.4253/wjge.v7.i11.1023 PMC4549659

[6] Nagino M, Hirano S, Yoshitomi H, Aoki T, Uesaka K, Unno M, et al. Clinical practice guidelines for the management of biliary tract cancers 2019: the 3rd English edition. J Hepatobiliary Pancreat Sci. 2021;28(1):26–54. 33259690 10.1002/jhbp.870

[7] Katabathina VS, Dasyam AK, Dasyam N, Hosseinzadeh K. Adult bile duct strictures: role of MR imaging and MR cholangiopancreatography in characterization. Radiographics. 2014;34(3):565–86. 24819781 10.1148/rg.343125211

[8] Barish MA, Yucel EK, Ferrucci JT. Magnetic resonance cholangiopancreatography. N Engl J Med. 1999;341(4):258–64. 10413739 10.1056/NEJM199907223410407

[9] Sundaram KM, Morgan MA, Itani M, Thompson W. Imaging of benign biliary pathologies. Abdom Radiol (NY). 2023;48(1):106–26. 35201397 10.1007/s00261-022-03440-5

[10] Sodickson A, Mortele KJ, Barish MA, Zou KH, Thibodeau S, Tempany CM. Three-dimensional fast-recovery fast spin-echo MRCP: comparison with two-dimensional single-shot fast spin-echo techniques. Radiology. 2006;238(2):549–59. 16436816 10.1148/radiol.2382032065

[11] Seo N, Park MS, Han K, Kim D, King KF, Choi JY, et al. Feasibility of 3D navigator-triggered magnetic resonance cholangiopancreatography with combined parallel imaging and compressed sensing reconstruction at 3T. J Magn Reson Imaging. 2017;46(5):1289–97. 28295827 10.1002/jmri.25672

[12] Lustig M, Donoho D, Pauly JM. Sparse MRI: the application of compressed sensing for rapid MR imaging. Magn Reson Med. 2007;58(6):1182–95. 17969013 10.1002/mrm.21391

[13] Morimoto D, Hyodo T, Kamata K, Kadoba T, Itoh M, Fukushima H, et al. Navigator-triggered and breath-hold 3D MRCP using compressed sensing: image quality and method selection factor assessment. Abdom Radiol (NY). 2020;45(10):3081–91. 31925493 10.1007/s00261-020-02403-y

[14] Worters PW, Sung K, Stevens KJ, Koch KM, Hargreaves BA. Compressed-sensing multispectral imaging of the postoperative spine. J Magn Reson Imaging. 2013;37(1):243–8. 22791572 10.1002/jmri.23750 PMC3473176

[15] Nagata S, Goshima S, Noda Y, Kawai N, Kajita K, Kawada H, et al. Magnetic resonance cholangiopancreatography using optimized integrated combination with parallel imaging and compressed sensing technique. Abdom Radiol (NY). 2019;44(5):1766–72. 30659308 10.1007/s00261-018-01886-0

[16] Tsai CM, Nishimura DG. Reduced aliasing artifacts using variable-density k-space sampling trajectories. Magn Reson Med. 2000;43(3):452–8. 10725889 10.1002/(sici)1522-2594(200003)43:3<452::aid-mrm18>3.0.co;2-b

[17] Cook RL. Stochastic sampling in computer graphics. ACM Trans Graphics (TOG). 1986;5(1):51–72.

[18] Gdaniec N, Eggers H, Bornert P, Doneva M, Mertins A. Robust abdominal imaging with incomplete breath-holds. Magn Reson Med. 2014;71(5):1733–42. 23818230 10.1002/mrm.24829

[19] Plonka G, Ma J. Curvelet-wavelet regularized split Bregman method for compressed sensing. Int J Wavelets Multiresolut Inf Process. 2011;9:79–110.

[20] Kondo S, Nakamura Y, Higaki T, Nishihara T, Takizawa M, Shirai T, et al. Utility of wavelet denoising with geometry factor weighting for gadoxetic acid-enhanced hepatobiliary-phase MR imaging. Magn Reson Med Sci. 2023;22(2):241–52. 35650028 10.2463/mrms.mp.2022-0041 PMC10086400

[21] Takata T, Ichikawa K, Mitsui W, Hayashi H, Minehiro K, Sakuta K, et al. Object shape dependency of in-plane resolution for iterative reconstruction of computed tomography. Phys Med. 2017;33:146–51. 28089191 10.1016/j.ejmp.2017.01.001

[22] Klessen C, Asbach P, Kroencke TJ, Fischer T, Warmuth C, Stemmer A, et al. Magnetic resonance imaging of the upper abdomen using a free-breathing T2-weighted turbo spin echo sequence with navigator triggered prospective acquisition correction. J Magn Reson Imaging. 2005;21(5):576–82. 15834908 10.1002/jmri.20293

[23] Likert R. A technique for the measurement of attitudes. Arch Psychol. 1932;140:55.

[24] Phelps AS, Naeger DM, Courtier JL, Lambert JW, Marcovici PA, Villanueva-Meyer JE, et al. Pairwise comparison versus Likert scale for biomedical image assessment. AJR Am J Roentgenol. 2015;204(1):8–14. 25539230 10.2214/AJR.14.13022

[25] Tatsugami F, Higaki T, Kawashita I, Fukumoto W, Nakamura Y, Matsuura M, et al. Improvement of spatial resolution on coronary CT angiography by using super-resolution deep learning reconstruction. Acad Radiol. 2023;30(11):2497–504. 36681533 10.1016/j.acra.2022.12.044

[26] Svanholm H, Starklint H, Gundersen HJ, Fabricius J, Barlebo H, Olsen S. Reproducibility of histomorphologic diagnoses with special reference to the kappa statistic. APMIS. 1989;97(8):689–98. 2669853 10.1111/j.1699-0463.1989.tb00464.x

[27] Kim KW, Lee JM, Jeon YS, Kang SE, Baek JH, Han JK, et al. Free-breathing dynamic contrast-enhanced MRI of the abdomen and chest using a radial gradient echo sequence with K-space weighted image contrast (KWIC). Eur Radiol. 2013;23(5):1352–60. 23187728 10.1007/s00330-012-2699-4

[28] Kajita K, Goshima S, Noda Y, Kawada H, Kawai N, Okuaki T, et al. Thin-slice free-breathing pseudo-golden-angle radial stack-of-stars with gating and tracking T1-weighted acquisition: an efficient gadoxetic acid-enhanced hepatobiliary-phase imaging alternative for patients with unstable breath holding. Magn Reson Med Sci. 2019;18(1):4–11. 29526882 10.2463/mrms.mp.2017-0173 PMC6326769

[29] Chandarana H, Block TK, Rosenkrantz AB, Lim RP, Kim D, Mossa DJ, et al. Free-breathing radial 3D fat-suppressed T1-weighted gradient echo sequence: a viable alternative for contrast-enhanced liver imaging in patients unable to suspend respiration. Invest Radiol. 2011;46(10):648–53. 21577119 10.1097/RLI.0b013e31821eea45

[30] Yoon JH, Lee SM, Kang HJ, Weiland E, Raithel E, Son Y, et al. Clinical feasibility of 3-dimensional magnetic resonance cholangiopancreatography using compressed sensing: comparison of image quality and diagnostic performance. Invest Radiol. 2017;52(10):612–9. 28448309 10.1097/RLI.0000000000000380

[31] Matsuyama T, Ohno Y, Yamamoto K, Ikedo M, Yui M, Furuta M, et al. Comparison of utility of deep learning reconstruction on 3D MRCPs obtained with three different k-space data acquisitions in patients with IPMN. Eur Radiol. 2022;32(10):6658–67. 35687136 10.1007/s00330-022-08877-2

[32] Zhang Y, Peng W, Xiao Y, Ming Y, Ma K, Hu S, et al. Rapid 3D breath-hold MR cholangiopancreatography using deep learning-constrained compressed sensing reconstruction. Eur Radiol. 2023;33(4):2500–9. 36355200 10.1007/s00330-022-09227-y

[33] Kiriyama S, Kozaka K, Takada T, Strasberg SM, Pitt HA, Gabata T, et al. Tokyo guidelines 2018: diagnostic criteria and severity grading of acute cholangitis (with videos). J Hepatobiliary Pancreat Sci. 2018;25(1):17–30. 29032610 10.1002/jhbp.512

