# Supplementary figures and images for: Utility of under-sampled scans with iterative reconstruction and high-frequency preserving transform for high spatial resolution magnetic resonance cholangiopancreatography
Source: Jpn J Radiol. 2024 Nov 5;43(3):463–71. doi: 10.1007/s11604-024-01688-z (PMC11868363; doi:10.1007/s11604-024-01688-z)

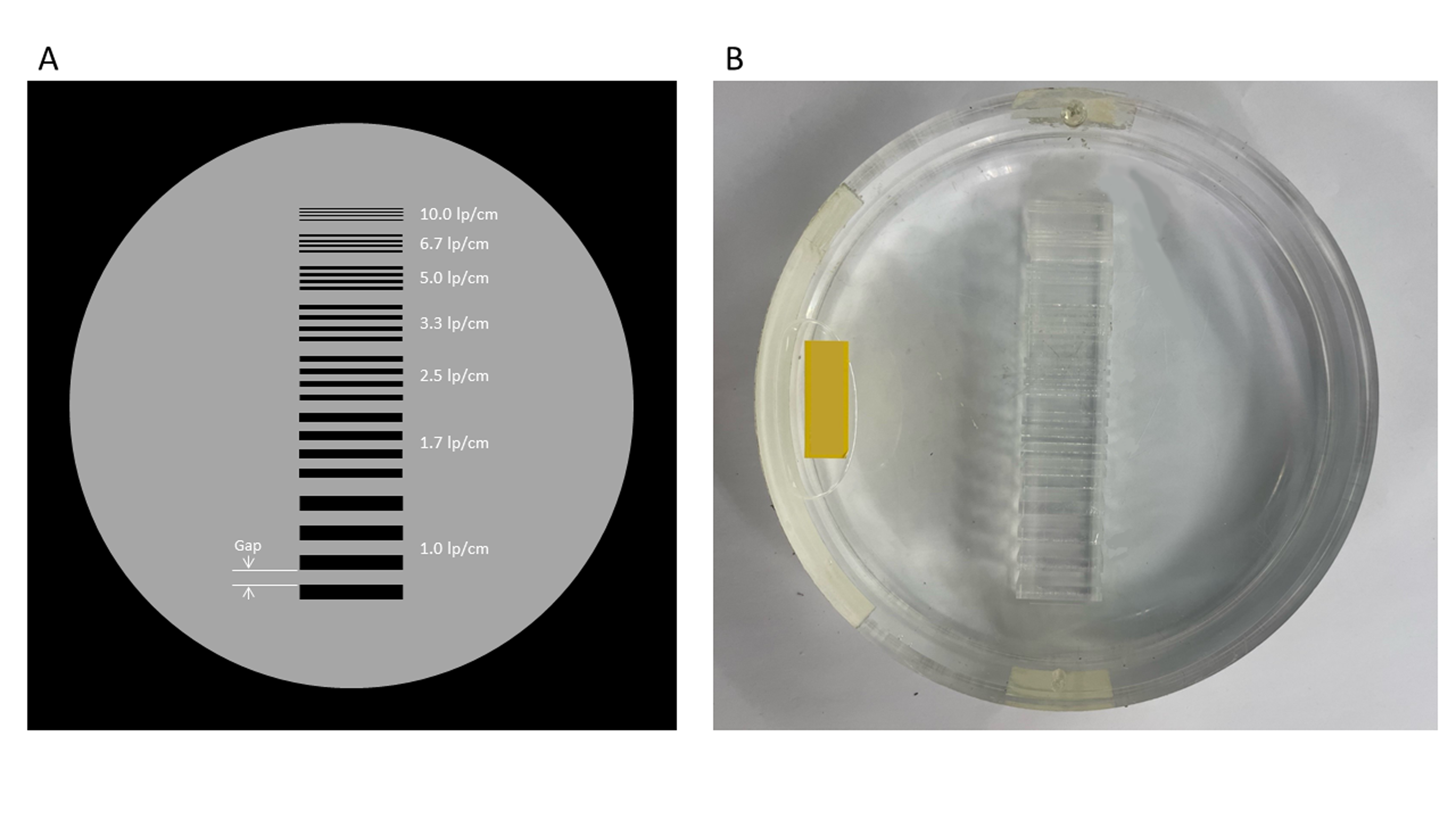

Supplement: Supplementary file 1 — Supplementary Fig. 1 Design (A) and actual photo (B) of our slit phantom. The gauges were filled with saline and the area around the gauges was filled with an NiCl2 solution [file 11604_2024_1688_MOESM1_ESM.tif]

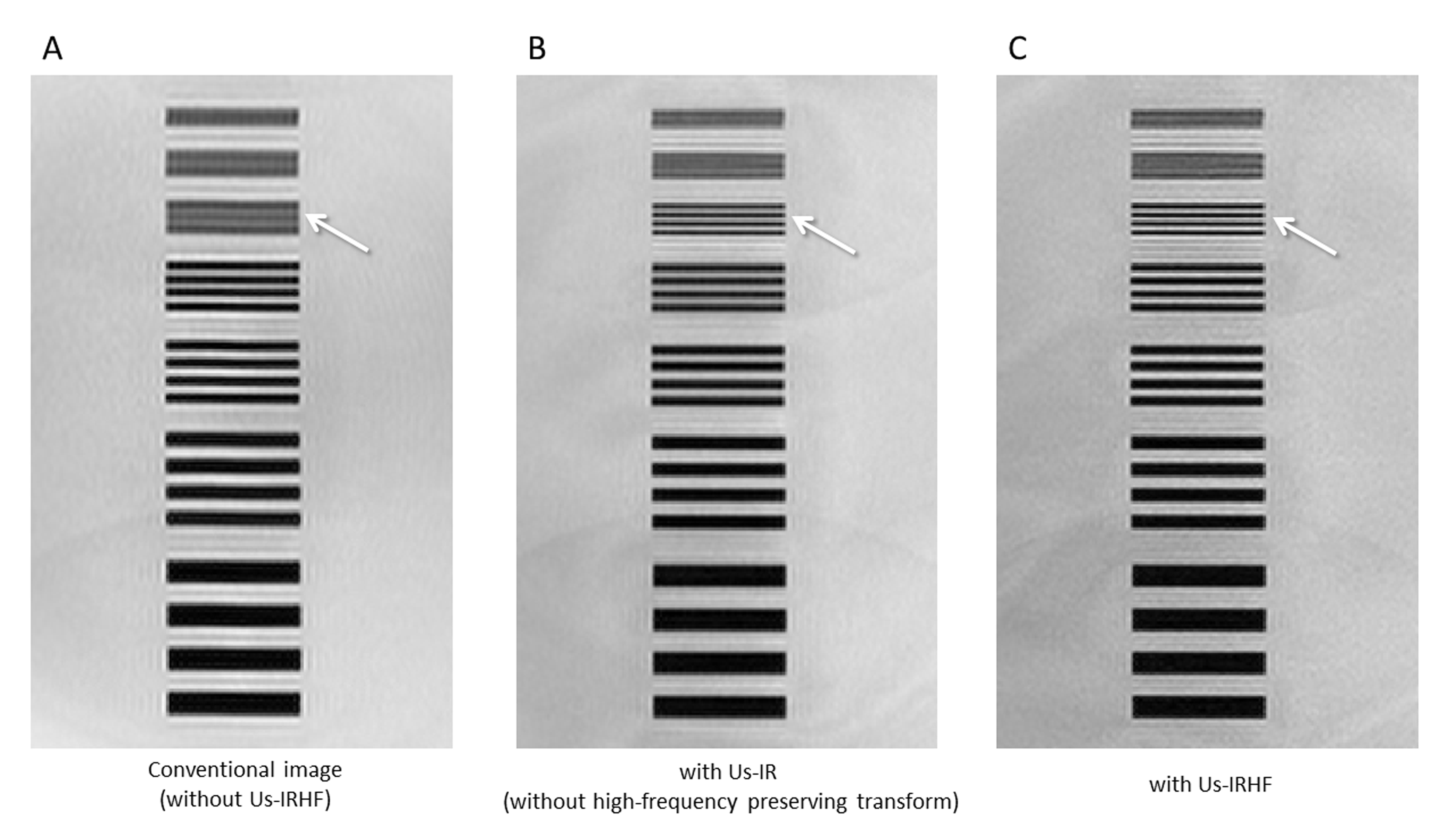

Supplement: Supplementary file 2 — Supplementary Fig. 2 Phantom images obtained with conventional- (A), Us-IR- (without high-frequency preserving transform) (B), and Us-IRHF scanning (with high-frequency preserving transform) (C). The margin of the 5.0 lp/cm gauge (arrows) was separated visually on (B) and (C), but not on (A) [file 11604_2024_1688_MOESM2_ESM.tif]

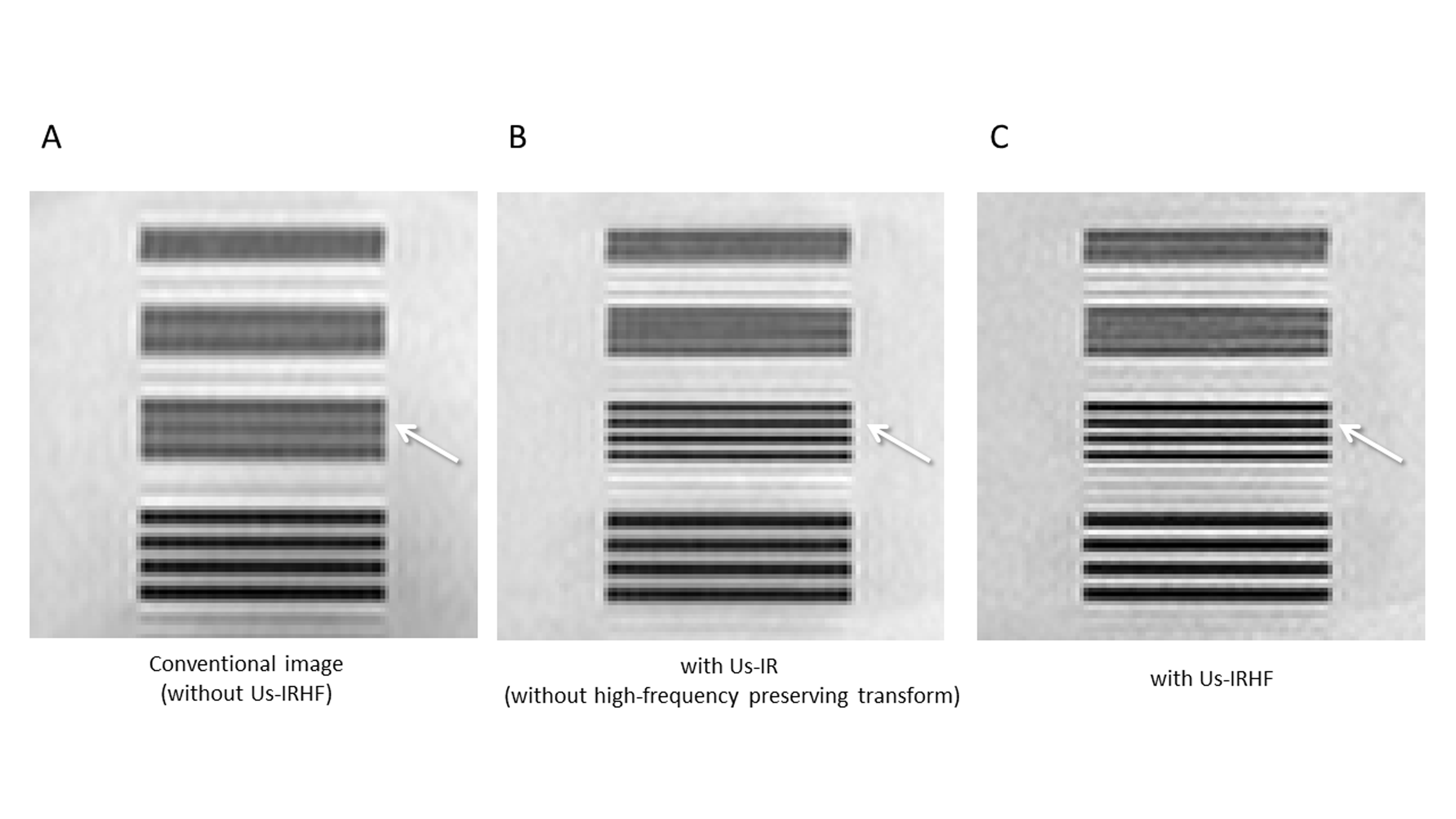

Supplement: Supplementary file 3 — Supplementary Fig. 3 Magnification (A–C) of images shown in Supplementary Fig. 2. The margin of the 5.0 lp/cm gauge (arrows) was separated most clearly on (C) [file 11604_2024_1688_MOESM3_ESM.tif]

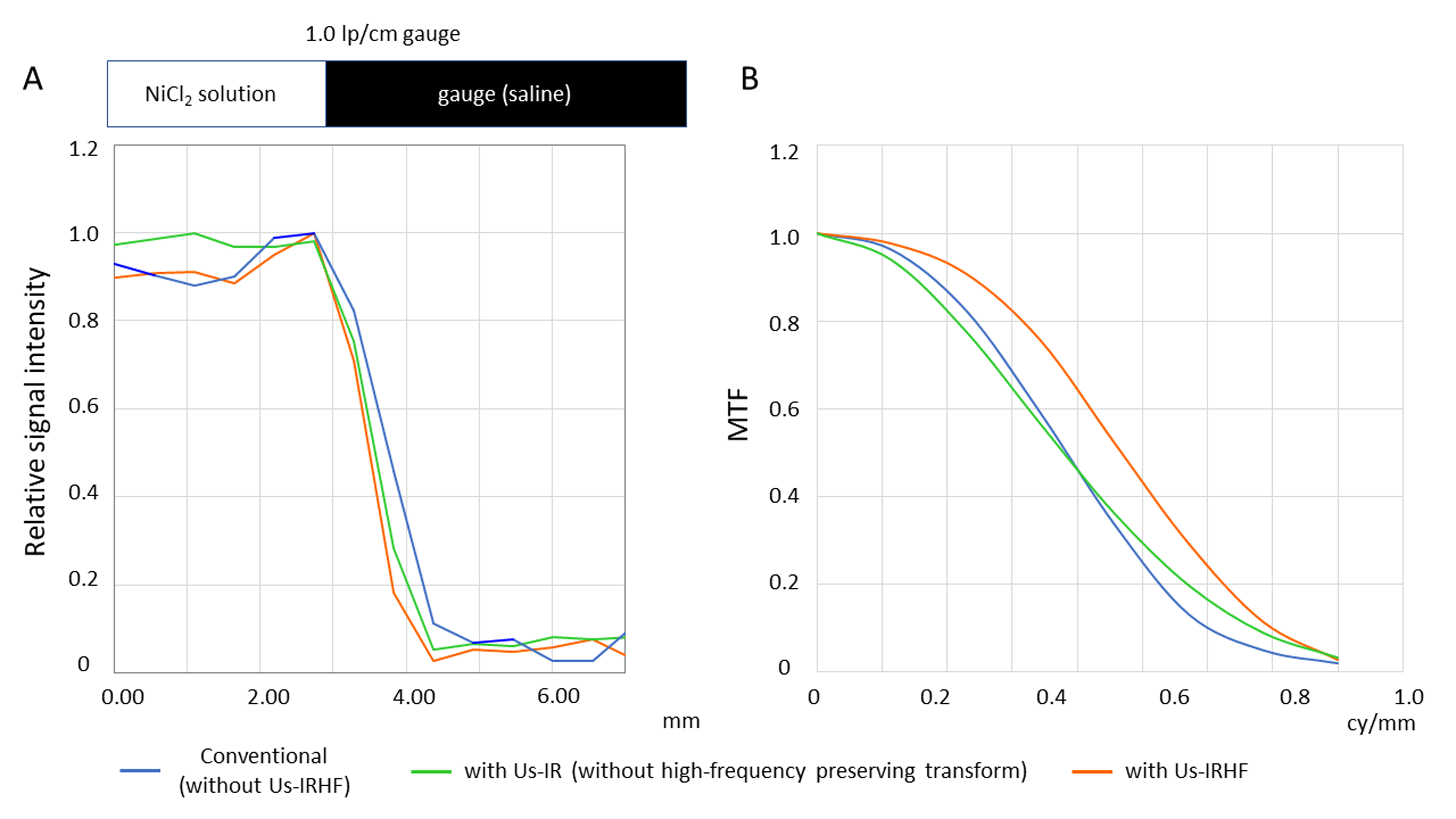

Supplement: Supplementary file 4 — Supplementary Fig. 4 Profile curve (A) and MTF (B) for the 1.0 lp/cm gauge obtained with conventional-, Us-IR- and Us-IRHF image [file 11604_2024_1688_MOESM4_ESM.tif]
