# Supplementary material for: Utility of under-sampled scans with iterative reconstruction and high-frequency preserving transform for high spatial resolution magnetic resonance cholangiopancreatography
Source: Jpn J Radiol. 2024 Nov 5;43(3):463–71. doi: 10.1007/s11604-024-01688-z (PMC11868363; doi:10.1007/s11604-024-01688-z)
Supplement: Supplementary file 5 — Supplementary Table 1 Qualitative scores on U+MRCP and U-MRCP images of each of three readers [file 11604_2024_1688_MOESM5_ESM.docx]

Supplementary Table 1. Qualitative scores on U^+^MRCP and U^-^MRCP images of each of three readers

| Image quality scores | U^+^MRCP | | | U^-^MRCP | | |
| --- | --- | --- | --- | --- | --- | --- |
| Readers | 1 (KN) | 2 (YN) | 3 (KA) | 1 (KN) | 2 (YN) | 3 (KA) |
| MPD | 3.4 (1.0) | 3.4 (1.1) | 3.4 (1.1) | 3.0 (0.9) | 3.0 (0.9) | 3.0 (1.0) |
| CBD | 3.9 (0.9) | 3.8 (0.9) | 3.9 (0.9) | 3.6 (0.8) | 3.5 (0.8) | 3.5 (0.8) |
| LHD | 3.5 (0.9) | 3.5 (0.9) | 3.6 (0.9) | 3.2 (0.8) | 3.2 (0.9) | 3.2 (0.9) |
| RHD | 3.6 (1.0) | 3.5 (1.0) | 3.6 (1.0) | 3.1 (0.9) | 3.0 (0.9) | 3.2 (1.0) |
| Overall image quality | 3.8 (0.8) | 3.9 (0.8) | 3.9 (0.9) | 3.3 (0.8) | 3.3 (0.8) | 3.4 (1.1) |

Data are the mean (standard deviation).

MPD: main pancreatic duct, CBD: common bile duct, LHD: left hepatic duct, RHD: right hepatic duct
